# Generalising semantic category disambiguation with large lexical resources for fun and profit

**DOI:** 10.1186/2041-1480-5-26

**Published:** 2014-06-02

**Authors:** Pontus Stenetorp, Sampo Pyysalo, Sophia Ananiadou, Jun’ichi Tsujii

**Affiliations:** 1 Department of Computer Science, University of Tokyo, Tokyo, Japan; 2 School of Computer Science, University of Manchester, Manchester, UK; 3 National Centre for Text Mining, University of Manchester, Manchester, UK; 4 Microsoft Research Asia, Beijing, People’s Republic of China

**Keywords:** Semantic category disambiguation, Approximate string matching, Lexical resources, Named entity recognition, Domain adaptation, Freebase

## Abstract

**Background:**

Semantic Category Disambiguation (SCD) is the task of assigning the appropriate semantic category to given spans of text from a fixed set of candidate categories, for example Protein to “Fibrin”. SCD is relevant to Natural Language Processing tasks such as Named Entity Recognition, coreference resolution and coordination resolution. In this work, we study machine learning-based SCD methods using large lexical resources and approximate string matching, aiming to generalise these methods with regard to domains, lexical resources and the composition of data sets. We specifically consider the applicability of SCD for the purposes of supporting human annotators and acting as a pipeline component for other Natural Language Processing systems.

**Results:**

While previous research has mostly cast SCD purely as a classification task, we consider a task setting that allows for multiple semantic categories to be suggested, aiming to minimise the number of suggestions while maintaining high recall. We argue that this setting reflects aspects which are essential for both a pipeline component and when supporting human annotators. We introduce an SCD method based on a recently introduced machine learning-based system and evaluate it on 15 corpora covering biomedical, clinical and newswire texts and ranging in the number of semantic categories from 2 to 91.

With appropriate settings, our system maintains an average recall of 99% while reducing the number of candidate semantic categories on average by 65% over all data sets.

**Conclusions:**

Machine learning-based SCD using large lexical resources and approximate string matching is sensitive to the selection and granularity of lexical resources, but generalises well to a wide range of text domains and data sets given appropriate resources and parameter settings. By substantially reducing the number of candidate categories while only very rarely excluding the correct one, our method is shown to be applicable to manual annotation support tasks and use as a high-recall component in text processing pipelines. The introduced system and all related resources are freely available for research purposes at: https://github.com/ninjin/simsem.

## Background

**Figure 1:**
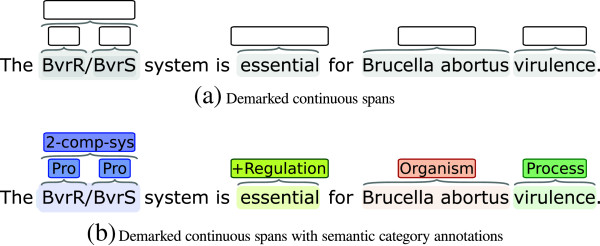
**Example of the prerequisite for our task setting, demarked continuous spans as seen in (a) and the output, semantic categories assigned to the input spans as seen in (b).** “2-comp-sys”, “Pro” and “+Regulation” are used as short-hands for “Two-component system”, “Protein” and “Positive regulation” respectively. Note the potential for partial overlap of different semantic categories as can be seen for the “Protein” and “Two-component system” annotations.

Semantic Category Disambiguation (SCD) is a key sub-task of several core problems in Natural Language Processing (NLP). SCD is of particular importance for Named Entity Recognition (NER), which conceptually involves two sub-tasks that must be solved: detecting entity mentions and determining to which semantic category a given mention belongs. SCD is concerned with the latter, the selection of the appropriate semantic category to assign for a given textual span from a set of candidate categories (Figure 1). Other tasks that SCD is relevant to include coreference and coordination resolution. In coreference resolution [1], coreferring mentions must share the same semantic category, and a method can thus exclude candidate mentions by having access to accurate semantic classifications. Also, by adding semantic information about the members of a coordinate clause, it is possible to resolve that the most likely meaning for a phrase such as “Tea or coffee and a sandwich” is “[[Tea or coffee] and a sandwich]” rather than “[[Tea] or [coffee and a sandwich]]” [2].

We recently demonstrated that high-performance SCD systems can be constructed using large-scale lexical resources and approximate string matching for several well-established data sets [3]. However, a number of questions regarding the applicability of these methods remain unanswered. First, this approach to SCD has only been extensively evaluated for biomedical texts, which raises the question whether the advances made for the biomedical domain can readily be carried over to other domains such as clinical and newswire texts. Second, state-of-the-art NER and SCD systems typically rely on lexical resources selected to suit the task being addressed [4,5] and one can thus expect performance to degrade if the system is moved to a new domain or language [6], but the magnitude of this effect for SCD has not been established. Third, while NER data sets are commonly annotated for short, non-embedded text spans such as person names or protein mentions, in a number of corpora annotations can cover long spans and be nested in complex structures [7]. We would expect such annotations to pose issues for lexical resource matching strategies that rely on a mapping between the resource and the span being classified.

**Figure 2:**
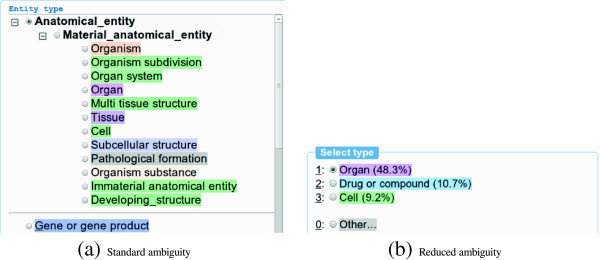
**Examples of entity type annotations from **[25]**, illustrating how the amount of visual and user-interface complexity (a) can be reduced using an SCD system (b).** The relevant text span being annotated in both figures is “heart” which should be assigned the ORGAN semantic category.

There are several practical applications that involve SCD, such as the assignment of labels such as those of ICD-10 [8] to documents and the production of annotations to train information extraction systems [9]. For any manual assignment task, there are cognitive limitations on the number of distinct categories a human annotator can process before falling victim to degrading performance [10]. Automated systems could thus assist annotators by limiting the number of categories presented to the user, excluding those that are clearly irrelevant; Figure 2 shows an illustration for a specific use-case. However, any annotation support system will be subject to close scrutiny, and an SCD system must thus have very high recall to avoid errors and rejection by users, while at the same time limiting the number of categories presented to the highest degree possible, even when the amount of training data is limited.

In this work we extend our initial study [11] of the applicability of SCD for annotation support and as a pipeline system component, investigating whether SCD can be generalised across domains and languages and the impact of lexical resource selection and differences in annotation criteria.

## Methods

This section discusses baseline methods, evaluation metrics, feature sets, models, corpora and lexical resources used for the experiments.

### Previous work and baseline methods

Although SCD is central to NER and several other NLP tasks, there have been relatively few in-domain studies investigating SCD as a stand-alone task. However, recently a few publications have investigated this task in isolation. Cohen et al. [12] presented a fast and reliable approach for associating a given textual span to one or several ontologies. The method was based on a set of manually crafted rules and achieved an accuracy ranging from 77.1% to 95.5% for determining the semantic category of a given annotation in a setting where each category was defined by reference to a domain ontology. In recent work, [3] we introduced a machine learning-based SCD method that employed approximate string matching [13] of continuous textual spans to several large-scale lexical resources. While the use of lexical resources such as dictionaries covering specific semantic categories is commonplace in state-of-the-art NER systems [4,5], approximate string matching was a novel aspect of the work. We evaluated the method on several data sets and achieved results ranging from 85.9% to 95.3% in accuracy. However, although the overall best-performing model in this study incorporated approximate string matching features, we failed to establish a clear systematic benefit of approximate, as opposed to strict, string matching for all data sets.

Since our goal here is to evaluate the performance of SCD for assisting other tasks such as manual text annotation, the approach of Cohen et al. has two limitations. First, it assumes that the semantic categories are defined by ontological resources and therefore it cannot be directly applied to annotation targets that do not match available ontological resources. Second, unlike our previously proposed approach, their approach does not provide ranking or classification confidence. Since this makes the method less suitable in a setting where it is necessary to dynamically adjust the number of suggested categories, as is the case for annotation support, for the present study we choose to extend our previous system.

### Task setting

We define an SCD task as follows: for a fixed set of candidate semantic categories, given a text and a continuous textual span in its context, assign the correct category to the span. Figure 1 illustrates the style of annotation and the possibility of overlapping and nested spans with different semantic categories. The SCD task set-up is related to both Word-sense Disambiguation [14] (WSD) and term grounding (or normalisation) [15], but there are several noteworthy differences. The spans considered in WSD are inherently internally ambiguous (for example “exploit” carrying the meaning of achievement, advantageous use, etc.), thus requiring the word sense to be mainly inferred by context. Further, SCD assumes a fixed set of categories, while in WSD the senses are normally different for each ambiguous word. In term grounding, entity mentions are to be mapped to unique identifiers, typically defined with reference to large resources such as Entrez Gene or Wikipedia, and each identifier represents only a small number of entities or just a single one. The key difference in this case is that as SCD is concerned with broader semantic categories, each covering a large number of entities, SCD methods can thus assume that the training data will contain numerous instances of each possible category.

In our previous work [3] we cast SCD as a (single-label) classification task, and Cohen et al. [12] considered it as a multi-label classification task. In this work we consider both the single-label classification setting as well as a setting where we allow the method to suggest any number of categories for a given span, in part analogously to beam search with a dynamic width beam [16]. Although in our data exactly one candidate category is correct for each span, this setting allows us to explore high-recall approaches while aiming to keep the number of suggestions to a minimum.

This setting matches our aim of reducing the cognitive burden on a human annotator who has to determine the correct answer among multiple suggestions and allows us to study how well an SCD system can estimate its own confidence when passing on suggestions to either a human annotator or another system.

### Metrics

For single-label classification, we report performance using accuracy, the fraction of cases where the system predicts the correct label. When allowing the system to suggest multiple categories, we measure recall and ambiguity reduction. Recall is the fraction of cases where the correct category is present among the suggested categories, and ambiguity is defined as the number of semantic categories suggested by the system. While both recall and (average) ambiguity give insight into the performance of the system, they are in a trade-off relation, similarly to how precision is to recall, and should ideally be combined into a single metric. To normalise the ambiguity metric with regard to the number of categories, we define (relative) ambiguity reduction as follows:

(1) $$\text{AmbiguityReduction}=\frac{|\text{Categories}|-\text{Ambiguity}}{|\text{Categories}|-1}$$

Here, we subtract one from the number of categories in the denominator to give the metric the same range as recall ([ 0.0,…,1.0]). We then straightforwardly combine average ambiguity reduction and recall into a harmonic mean.

We train our model and produce learning curves with data points using samples of [ 5*%*,10*%*,…,100*%*] of the training data. At each data point we take several random samples of the current data size and use the mean (*μ*) of the performance over the samples to compensate for possible sample variance. Results for each metric are provided as the mean of the data points of the learning curve, as is commonly done to approximate the Area Under the Curve (AUC).

### Feature sets and models

One of the primary differentiating factors between the machine learning models considered in our experiments are the feature sets applied in training each model. Our three baseline models are defined by the following feature sets: INTERNAL (INT.), a comprehensive set of NER-inspired features derived solely from the text span to be classified, GAZETTEER (GAZ.) features derived from strict string matching look-ups of the span in each of the applied lexical resources, and SIMSTRING (SIM.) features, representing an approximate matching variant of GAZETTEER calculated using the SimString approximate string matching library [13]. These feature sets are described in detail in our previous studies [3,17]. The three baseline methods are defined by the feature set combinations INTERNAL, INTERNAL+GAZETTEER, and INTERNAL+SIMSTRING, abbreviated as INT., INT.GAZ. and INT.SIM., respectively.

We extended our previous system described in [3] to allow it to determine the number of categories to propose to optimise recall and ambiguity reduction as follows. The machine learning method applied in the system [18] provides probabilistic outputs, which can be used as indicators of the confidence the method has for each category. The system considers the categories ordered highest-confidence first, and returns the smallest set of categories so that the sum of the confidences for the set is equal to or greater than a threshold value. This threshold becomes an additional parameter for the system, controlling the trade-off between ambiguity and recall. This will result in a number of suggestions ranging from 1 to the total number of categories in the data set. For example, for the categories and confidences [PROTEIN 90%, CHEMICAL 6%, ORGANISM 4% ] and the confidence threshold 95%, the system would suggest PROTEIN and CHEMICAL, but not ORGANISM. In our previous work, [11] we selected a threshold of 99.5% as this performed well for the evaluation on the development data sets, and we continued to use this threshold for our initial experiments here.

### Corpora

For evaluation, we initially included the six data sets used in [3], listed above the mid-line in Table 1. While our previous study found promising results for these data sets, they are all drawn from the biomedical domain, which left the generality of our method largely unsubstantiated. To argue that our method is applicable to other domains, we need to show this experimentally. To demonstrate the applicability of the method, it is also necessary to evaluate against corpora containing more semantic categories than the 17 covered by the EPI data set, the largest number in the previously considered resources. To widen our selection of annotated resources, we thus collected a total of nine additional corpora, listed below the mid-line in Table 1 and presented in the following.

**Table 1:** Corpora used for evaluation

**Name**	**Semantic categories**
Epigenetics and Post-Translational	17
Modifications corpus [35] (EPI)	
Infectious Diseases corpus [22] (ID)	16
Genia Event corpus [36] (GE)	11
Collaborative Annotation of a Large	4
Biomedical Corpus [37] (SSC)	
BioNLP/NLPBA 2004 Shared Task	5
corpus [38] (NLPBA)	
Gene Regulation Event Corpus [39] (GREC)	64 (6)
Multi-Level Event Extraction corpus [21] (MLEE)	52
GeneReg corpus [40] (GReg)	10
Gene Expression Text Miner corpus [41] (GETM)	3
BioInfer [7] (BI)	119 (97)
BioText [42] (BT)	2
CoNLL-2002 Shared Task corpus,	4
Spanish subset [20] (CES)	
CoNLL-2002 Shared Task corpus, Dutch	4
subset [20] (CNL)	
i2b2 Medication Challenge corpus [19] (I2B2)	6
OSIRIS corpus [43] (OSIRIS)	2

Parenthesised values signify the actual number of categories after performing pre-processing steps so as to not suffer from data sparseness (GREC conversion into SGREC[3]) or to compensate for ontological design decisions (BI). The mid-line indicates a cut-off between the above corpora used in previous work [3] and the corpora added to evaluate our approach for a variety of domains and covering a large set of semantic categories.

To extend the coverage of domains, we included the I2B2 corpus [19] and the CoNLL-2002 data sets for Spanish and Dutch NER [20]. I2B2 stems from the clinical domain which, while related to the biomedical domain, involves a different set of semantic categories (e.g. DOSAGE and MEDICATION). The CoNLL-2002 data sets are both from the newswire domain, largely unrelated to the previously considered domains, and additionally for languages other than English. They are thus expected to pose new challenges, in particular in regards to the lexical resources utilised. As mentioned above, the question is still open as to whether our approach scales to a set of categories larger than the 17 of the EPI corpus. To address this issue, we acquired the MLEE [21] and BI [22] corpora which contain 52 and 119 semantic categories each, representing increases of ∼ 3× and ∼ 7× respectively in the number of categories. Finally, we added four biomedical corpora not considered in previous work to increase the diversity of resources in this domain.

Following initial corpus selection, we performed some pre-processing for a number of the resources, as follows. After inspecting the annotation guidelines for the BI corpus, we found that a core assumption of our task setting was violated: mentions of entities of the three semantic categories GENE, PROTEIN and RNA would be marked using a single compound category (GENE, PROTEIN OR RNA) if they were not a participant of an event annotation. This is problematic for our experimental set-up since we do not seek to model whether targeted entity mentions participate in events. Thus, we collapsed all entries for GENE, PROTEIN and RNA into the single GENE, PROTEIN OR RNA category as a pre-processing step. Furthermore, BI allows for discontinuous span annotations, which also conflicts with the assumptions of our task setting. We thus merged all discontinuous spans into single spans, removing any duplicate spans that were created in the process. Finally, to compensate for an ontological decision to differentiate between state changes and processes (e.g. “Phosphorylate” compared to “Phosphorylation”) we merged all paired types into single categories. After these pre-processing steps had been carried out, we were left with 97 distinct semantic categories, a ∼ 6× increase compared to the largest number of categories considered in our previous study. We also performed some necessary, but less involved, pre-processing steps for some other corpora. In the case of BT, we removed the relational indicators for each span and used the two categories DISEASE and TREATMENT. For I2B2, we used the gold data annotated and released by the organisers of the shared task, leaving out the parts of the provided data submitted by shared task participants.

**Figure 3:**
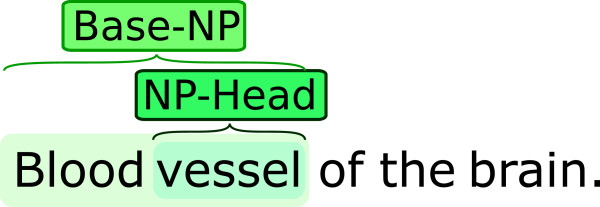
Example of sub-string components used to generate the NP-based features.

All the data sets were randomly separated into training, development and test sets consisting of 1/2, 1/4 and 1/4 of the annotations respectively. The test set was kept hidden during development and was only used to generate the final results. When reviewing annotation samples and guidelines for the nine additional corpora, we found some cases that we anticipated would be problematic for methods using our previously proposed feature sets. In particular, for compound noun-phrases (NPs) containing mentions of entities of several different semantic categories, the classifier could potentially be confused by matches to resources containing semantic categories unrelated to the entity referred to by the NP as a whole. As a concrete example, consider “Complex of fibrin and plasminogen”: the full span should be assigned the semantic category COMPLEX, while the semantic categories of “fibrin” and “plasminogen” are PROTEIN. To address such cases, we drew on the observation that the head word of a noun-phrase commonly determines the semantic category of a span. Specifically, we constructed a set of features employing a simple heuristic-based noun-phrase head-finding algorithm, extracting two span components of particular interest: the NP-head detected by the algorithm, and the Base-NP, approximated as the combination of the NP-head and all preceding text in the span (Figure 3). These subspans were used in feature generation to define an extended NP feature set: for the INTERNAL feature set, we added binary features representing the text of the NP-head and Base-NP, and for the GAZETTEER and SIMSTRING feature sets, we performed look-ups against all lexical resources using strict and approximate string matching respectively, in addition to the binary features for the text of the NP-head and Base-NP. We will discuss the impact of these features for the various data sets in the Results and discussion section.

### Lexical resources

As a starting point, we adopt the collection of 170 lexical resources first gathered in [3]. These are particularly suited for biomedical data as they were manually selected with this single domain in mind. Since it would be advantageous to use a general purpose collection of lexical resources rather than those selected for a specific domain, we also evaluate the data provided by the Freebase project as a source of general-purpose lexical resources. The Freebase knowledge base covers a wide range of domains, is multi-lingual in nature, and has recently been utilised for several NLP tasks [23,24]. Freebase is collaboratively curated by volunteers and contains millions of “statements”. However, not all of these are relevant to our experiments, as the knowledge base not only covers statements regarding semantic categories but also information such as user data. The project defines a set of 72 “Commons” categories that have passed several community standards and cover a wide array of topics such as ASTRONOMY, GEOGRAPHY, GAMES, etc. We created 72 lexical resources from the 15,561,040 unique entry names listed for these Freebase categories, referred to in the following as FB.

Even though Freebase is a general-purpose resource, we anticipated some issues with the granularity of the “Commons” categories. In particular, the MEDICINE and BIOLOGY categories do not make any distinction between, for example, DRUG and INFECTIOUS DISEASE, and ORGANISM and GENE, respectively. In order to allow for a fair comparison to the manually selected biomedical domain lexical resources, we constructed an additional set of resources where these two categories anticipated to be problematic were split into their sub-categories, giving a total of 175 lexical resources. This set is referred to as FBX in the following.

The GAZETTEER and SIMSTRING features are dependent on the choice of lexical resources, and we can thus create variants of these feature sets by using any of the above-mentioned sets of lexical resources. For our experiments, we also defined in addition to the “basic” variant using the 170 biomedical domain resources four models based on the GAZETTEER and SIMSTRING in combination with the FB and FBX sets.

## Results and discussion

**Figure 4:**
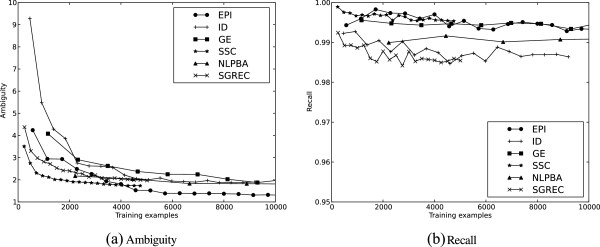
Learning curves for ambiguity (a) and recall (b) for our initial ambiguity experiments.

This section introduces and discusses the experimental outcomes. The experimental results are summarised in Figure 4, Table 2 and Additional file 1: Table S1. We first investigate how our baseline models perform in regards to ambiguity reduction and recall on the subset of corpora used in our previous work. Next, we proceed to evaluate how the same models perform for additional data sets, focusing on performance for resources with large numbers of semantic categories and those from domains which are either different but related (clinical) or largely unrelated (newswire) to the biomedical domain. We then evaluate the impact of utilising different lexical resources and evaluate the effectiveness of our proposed NP feature set. Lastly, we consider the effects of tuning the threshold parameter that controls the trade-off between ambiguity and recall.

**Table 2:** **Results for the BT, GETM, I2B2 and OSIRIS data sets using the Int.NP.Sim. model with a confidence threshold of 95% for mean ambiguity reduction (*μAmb.Red.*), mean recall (*μ Recall*), and the harmonic mean of mean ambiguity reduction and recall (*H *** **(*μAmb.Red., μRecall *))**

**Data set**	***μ A m b . R e d .***	***μ R e c a l l***	***H*** **( *μ A m b . R e d . *, *μ R e c a l l *)**
BT	78.00/+34.00	99.54/-00.31	87.46/+26.38
GETM	88.50/+32.50	99.99/-00.01	93.89/+22.10
I2B2	77.60/+42.60	98.14/-01.50	86.67/+34.87
OSIRIS	78.00/+42.00	99.79/-00.21	87.56/+34.62

The relative values are compared to the same model using a confidence threshold of 99.5%.

### Initial evaluation on biomedical corpora

For our initial investigations, we use the six corpora applied in our previous study [3]. Figures 4a and 4b show the lower end of the learning curves for ambiguity and recall, and the results for the different evaluation metrics are given in the boxed upper left corners in Additional file 1: Table S1.

We observe that the SIMSTRING model outperforms other baseline models in almost all cases where there are non-trivial differences between the different models. We thus focus primarily on the SIMSTRING model in the remainder of the evaluation. Our results are promising for both the ambiguity and recall metrics. Ambiguity quickly drops to a manageable level of 2–3 remaining categories for all corpora (Figure 4a), and the reduction in the number of semantic categories is on average 60% over the data sets (Additional file 1: Table S1c). The reduction is most prominent for EPI, where the number of categories is reduced by ∼95% even for the smallest training set size considered. The positive results for ambiguity reduction are achieved without compromising recall, which stays consistently around or above ∼99% for all data sets (Figure 4b and Additional file 1: Table S1d). This level is expected to be acceptable even for comparatively demanding users of the system. In summary, we find that for a number of biomedical domain data sets the proposed approach is capable of notably reducing the number of proposed semantic categories while maintaining a very high level of recall and that our SIMSTRING model outperforms other baseline models.

### Impact of data set domain and number of categories

We next extend our evaluation to the additional nine corpora incorporated in this study. As this gives 15 corpora in total, instead of considering performance metrics and learning curves in detail for each, we will below focus primarily on the summary results in Additional file 1: Tables S1a and S1b, giving accuracy and the harmonic mean of ambiguity reduction and recall. Among the nine additional data sets, CES, CNS and I2B2 are of particular interest regarding the ability of the approach to generalise to new domains; the former two are for languages different from English and from the newswire domain, a common focus of NLP studies, and the latter from the clinical domain. Likewise, the MLEE and BI data sets, containing 52 and 97 semantic categories respectively, are suited for evaluating the ability of the approach to generalise to tasks involving a large amount of semantic categories.

We first note that the SIMSTRING model performs well for all metrics for the biomedical domain MLEE, GREG and BI data sets. However, we observe several instances of reduced performance with respect to the results of the initial experiments for corpora of various domains. For the newswire domain CES and CNL data sets, we find somewhat reduced accuracy and a low harmonic mean. The biomedical domain GETM, BT and OSIRIS corpora and the clinical domain I2B2 corpus show high accuracy, but share the low harmonic mean performance of the CES and CNL data sets. In all cases the poor results in terms of the harmonic mean of ambiguity reduction and recall is due to low ambiguity reduction; recall remains high in all instances, reaching a full 100% in numerous cases (Additional file 1: Table S1d). This suggests that the method may have problems with its optimisation target when the number of categories is small, a property shared by all the above resources, overemphasising recall over ambiguity. Additionally, for the out-of-domain data sets it is probable that our selection of lexical resources is a poor fit, a possibility evaluated specifically in the next section.

In regards to data sets containing large sets of semantic categories, rather surprisingly both the MLEE and BI data sets appear to pose little challenge to our approach, even though they both contain more than three times the number of categories considered previously. These results suggest that, somewhat counter to expectation, the method appears to generalize well to large numbers of semantic categories, but poorly to small numbers of semantic categories.

### Lexical resource dependence

The poor performance for the Spanish and Dutch newswire corpora CES and CNL could potentially be explaned by a mismatch between the data sets and the applied lexical resources: the lexical resources originally used in [3] were collected specifically for the biomedical domain, and using only English resources. This hypothesis is supported by the observation that the models relying on lexical resources, SIMSTRING and GAZETTEER, performed poorly for these data sets, barely outperforming or performing slightly worse than the strong baseline of the INTERNAL model that does not utilise any lexical resources. To test the hypothesis, we created new SIMSTRING and GAZETTEER model variants using the Freebase-based lexical resources FB and FBX. These are denoted in Additional file 1: Table S1 by a trailing parenthesis following the model name that contains the resource name (e.g. “INT.SIM. (FB)”).

If we at first only consider the results of the FB-based models, we observe a considerable increase in performance for the CES and CNL data sets by approximately 4–5% points in mean accuracy and approximately 12–20% points in harmonic mean for the SIMSTRING model (Additional file 1: Table S1a and Additional file 1: Table S1b). This effect is most likely due to named entities annotated in these corpora, such as company names, person names, and locations, now being listed in the lexical resources and serving as strong features. An interesting observation is that although both the SIMSTRING and GAZETTEER models employ the same lexical resources, the performance increase for the SIMSTRING model greatly surpasses that of the GAZETTEER model. This result is largely analogous to what we have previously demonstrated for the biomedical domain, and suggests that the benefits of approximate string matching generalise also to the newswire domain and across languages.

Although the effect of using the “FB” version of the Freebase data is positive for the CES and CNL data sets, there is a notable drop in performance across the board for nearly all other data sets. At this point we should remember that we have anticipated that the Freebase “Commons” categories may be of limited value for specific domains due to their coarse granularity. We thus now also consider the results of the FBX-based models that give a finer granularity for the MEDICINE and BIOLOGY categories. For SIMSTRING, using FBX as opposed to FB raises the average accuracy over the data sets from 86.55% to 87.72% and the average harmonic mean score from 60.40% to 64.79%. Further, SIMSTRING is shown to benefit more than the strict string matching model GAZETTEER, which fails to realise a clear benefit from FBX as compared to FB. However, for the biomedical domain corpora, performance remains considerably lower than when using in-domain resources even for FBX.

These results confirm the expectation that the performance of the approach is strongly dependent on the choice of lexical resources, and suggest that while the large, general-purpose resource Freebase can be used to derive lexical resources applicable across domains, it cannot match the benefits derived from using targeted resources curated by specialists in the domain relevant to the corpus.

### Impact of noun-phrase head features

As noted in the introduction of the additional corpora, we were concerned that annotated spans of text that cover mentions of entities of multiple semantic categories may cause difficulties for our approach. This is in part due to our feature sets being inspired by features employed by NER systems, which frequently target short spans of text involving only single mentions of entities, such as proper names. To address this issue, we introduced the NP extensions of the feature sets of each model. In this section, we present results on the effectiveness of these features.

We find that GAZETTEER and SIMSTRING benefit from the introduction of the NP features, while INTERNAL shows mixed results depending on the metric. Interestingly, while GAZETTEER gains an average 0.60% points for accuracy and 6.39% points for the harmonic mean, the respective gains are lower for SIMSTRING, at 0.46% points and 4.51% points. Following from what we have observed previously, we would expect that if approximate string matching is more beneficial than strict matching on the level of the whole string, it would also be so on subsets of the same string. A possible explanation is that while the GAZETTEER model previously had no access to any substring matches in the lexical resources, the approximate string matching model could make some use of this information even before the introduction of the NP features. Thus, it is possible that in allowing matches against smaller regions of a given span, the use of approximate string matching to some extent relieves the need to perform detailed language-specific processing such as head-finding.

This evaluation demonstrated that the NP features are effective for the GAZETTEER and SIMSTRING models, with their addition to the SIMSTRING baseline feature set producing a model that outperforms all models in our previous work for a majority of the data sets for both the accuracy and harmonic mean metrics. The resulting model, INT.NP.SIM., is our best model as-of-yet for the SCD task.

### Impact of confidence threshold parameter

Until now we have not addressed the low performance in terms of ambiguity reduction for the GETM, BT, I2B2 and OSIRIS data sets. These are from the biomedical and clinical (I2B2) domains, but share the property of involving only a small number of semantic categories: three in GETM and two in the others. One parameter we kept fixed throughout experiments was the confidence threshold that controls the number of suggestions proposed by our system and the trade-off between ambiguity and recall. To investigate whether the setting of this parameter could account for the low performance for these resources, we lower the threshold from the value 99.5%, chosen based on experiments on the corpora used in our previous work [11], and instead use a threshold of 95.0%. This choice is motivated by a set of preliminary experiments on the development portions of all data sets. We then performed additional evaluation on the four above-mentioned corpora that had shown poor performance. We can observe that, as expected, performance in terms of ambiguity improves greatly (Table 2), roughly doubling in absolute terms. Further, this improvement is achieved while recall is preserved at a level of 98% or higher for all four data sets. In hindsight, this behaviour could be expected on the basis of our observation of close to perfect recall for the primary experiments for these four data sets.

This experiment shows that while a high threshold can cause the system to err on the side of recall and fail to produce a notable reduction in ambiguity for corpora with a low number of semantic categories, with an appropriate setting of the threshold parameter it is possible to achieve both high recall and a clear reduction in ambiguity also for such data sets.

## Conclusions and future work

We studied machine learning-based Semantic Category Disambiguation (SCD) methods using large lexical resources and approximate string matching, focusing on the ability of these SCD approaches to generalise to new corpora, domains, and languages, their dependence on factors such as the choice of lexical resources, and their applicability for annotation support tasks and as components in pipeline systems. Adapting an existing SCD method to a task setting allowing the system to suggest multiple candidates, we observed that performance is dependent on the choice and granularity of lexical resources and that resources with a low number of semantic categories and annotations involving mentions of multiple entities posed specific challenges for the method. We demonstrated how these issues could be addressed and were able to show that a 65% average reduction in the number of candidate categories can be achieved while maintaining average recall at 99% over a set of 15 corpora covering biomedical, clinical and newswire texts. We find these numbers very promising for the applicability of our system and will seek to integrate it as a component for other systems to further verify these results.

In future work, we hope to address a number of remaining questions. First, it should be verified experimentally that our primary metric, the harmonic mean of ambiguity and recall, represents a reasonable optimisation target for SCD applications such as annotation support. By varying the trade-off between ambiguity reduction and recall and measuring the impact on actual human annotation time [25], we could empirically study the relationship between ambiguity and recall for a given task. Furthermore, as we could observe in our lexical resource experiments, the optimal composition of lexical resources is dependent on the data set. While we could have manually constructed a new collection of lexical resources to cover all the domains in our experiments, this ad-hoc processes would potentially have to be repeated for each new data set we apply our method to. Instead, we propose to aim to automatically select the set of lexical resources optimal for each data set, which we believe to be more likely to result in long-term benefits and to allow our method to be beneficially applied to novel tasks. By integrating automatic lexical resource construction and confidence parameter selection, we hope to be able to create a general-purporse SCD method applicable across tasks and domains without the need for user intervention.

The system used in this study as well as other resources are freely available for research purposes at https://github.com/ninjin/simsem.

## Availability of code, corpora and lexical resources

This section covers the availability and sources for the code, corpora and lexical resources used in this work. In addition to assuring that those who have provided resources essential to this study are properly acknowledged, it aims to assist in the replication of the experiments presented in this paper.

The code used for the experiments is available under a permissive license from https://github.com/ninjin/simsem. The lexical resources used were Freebase, provided by Google and retrieved from https://developers.google.com/freebase/data on February the 9th of 2012, along with the 10 resources used to create dictionaries in [3], namely the Gene Ontology [26], the Protein Information Resource [27], the Unified Medical Language System (UMLS) [28], Entrez Gene [29], an Automatically generated dictionary [30], Jochem [31], the Turku Event Corpus [32], Arizona Disease Corpus [33], LINNAEUS Dictionary [34] and Webster’s Second International Dictionary from 1934 (included in /usr/share/dict/web2 in the FreeBSD 8.1-RELEASE). All of the above resources apart from UMLS are freely available for research purposes without restrictions. In UMLS, which to the best of our knowledge is the largest collection of biomedical lexical resources to-date, some of the component resources are restricted even for research usage. Please see the UMLS license for further details.

For our experiments we used the corpora originally used in [3]. These were: the Epigenetics and Post-Translational Modifications corpus [35], the Infectious Diseases corpus [22], the Genia Event corpus [36], the Collaborative Annotation of a Large Biomedical Corpus [37], the BioNLP/NLPBA 2004 Shared Task corpus [38] and the Gene Regulation Event Corpus [39]. For this work we also used the following corpora: the Multi-Level Event Extraction corpus [21], the GeneReg corpus [40], the Gene Expression Text Miner corpus [41], BioInfer [7], BioText [42], the Spanish and Dutch subsets of the CoNLL-2002 Shared Task corpus [20], the i2b2 Medication Challenge corpus (I2B2) [19] and the OSIRIS corpus [43]. The above corpora are readily available for research purposes with the exception of the I2B2 corpus, which due to its clinical nature does not allow for redistribution and/or exposure beyond researchers that have been explicitly authorised to utilise the data.

## Abbreviations

The followings abbreviations were used and introduced in this article.; NER: Named entity recognition; NLP: Natural language processing; SCD: Semantic category disambiguation; WSD: Word sense disambiguation.

## Competing interests

The authors declare that they have no competing interests.

## Authors’ contributions

PS and SP conceived of the methods and PS carried out the experiments and wrote the manuscript. SA and JT provided coordination and supervision of the overall research. All authors read and approved the final version of the manuscript.

## Supplementary Material

Additional file 1 **Table S1 Result tables for all data sets and models.** The boxed results in the upper left corner signifies the results from [11], while the unboxed results are additions for the extension of the original paper. The best score(s) for each data set are underlined and scores which are not statistically significantly different from the best result(s) with a P-value of 5% when using Fisher’s exact test are *italicised*[3]. Click here for file
