# Supplementary material for: Generalising semantic category disambiguation with large lexical resources for fun and profit
Source: J Biomed Semantics. 2014 Jun 2;5:26. doi: 10.1186/2041-1480-5-26 (PMC4107982; doi:10.1186/2041-1480-5-26)
Supplement: Additional file 1 — Table S1 Result tables for all data sets and models. The boxed results in the upper left corner signifies the results from [11], while the unboxed results are additions for the extension of the original paper. The best score(s) for each data set are underlined and scores which are not statistically significantly different from the best result(s) with a P-value of 5% when using Fisher’s exact test are italicised[3]. [file 2041-1480-5-26-S1.pdf]

$\mu Accuracy$	EPI	ID	GE	NLPBA	SSC	SGREC	MLEE	GREG	GETM	BI	BT	CES	CNL	I2B2	OSIRIS	μ
Int. [3]	*92.50*	*91.28*	*94.55*	91.64	81.71	82.04	78.68	92.75	*93.34*	80.87	84.92	78.36	79.00	76.46	80.25	85.22
Int.Gaz. [3]	93.53	92.12	94.66	91.80	89.32	82.89	*81.74*	*94.30*	*93.56*	81.66	84.92	78.77	79.46	79.48	*94.92*	87.54
Int.Sim. [3]	*93.29*	*92.07*	*94.36*	91.86	*92.03*	*83.95*	*83.20*	*94.08*	96.26	81.78	*94.89*	78.06	78.20	*87.34*	*96.02*	89.16
Int.NP.	*92.38*	90.35	*94.51*	*92.22*	81.63	82.67	78.78	92.59	*92.97*	81.47	87.54	78.29	79.02	76.92	81.66	85.53
Int.Gaz. (FB)	*92.58*	*91.17*	*94.58*	91.64	82.20	82.12	79.06	92.84	*93.36*	80.85	84.94	*81.14*	*82.67*	76.50	80.35	85.73
Int.Gaz. (FBX)	*92.57*	*91.15*	*94.58*	91.61	82.81	82.15	79.10	92.83	*93.36*	80.88	84.96	*81.12*	*82.60*	76.53	80.27	85.77
Int.NP.Gaz.	*93.44*	*91.71*	*94.63*	91.94	89.66	83.38	*82.75*	94.40	*93.51*	82.56	90.23	78.85	79.51	82.79	92.71	88.14
Int.NP.Gaz. (FB)	*92.55*	*91.15*	*94.59*	91.65	82.19	82.24	78.94	92.89	*93.31*	81.08	84.54	81.10	*82.99*	76.69	80.07	85.73
Int.NP.Gaz. (FBX)	*92.57*	*91.10*	*94.59*	91.64	82.95	82.29	79.02	92.88	*93.30*	81.16	84.89	81.10	*82.87*	76.71	80.13	85.81
Int.Sim. (FB)	*92.92*	*90.63*	*94.27*	91.61	82.44	82.27	78.03	92.40	*92.07*	80.71	87.31	82.23	*83.46*	80.03	87.92	86.55
Int.Sim. (FBX)	*93.07*	*90.71*	*94.37*	91.62	85.13	82.86	80.18	92.53	*93.05*	81.41	93.53	*82.12*	83.49	83.31	88.49	87.72
Int.NP.Sim.	*93.38*	*91.93*	*94.47*	92.53	92.12	85.82	84.11	*94.10*	*95.40*	84.73	95.88	77.50	78.00	88.17	96.12	89.62
Int.NP.Sim. (FB)	*93.05*	*90.56*	*94.09*	*92.15*	82.48	83.54	78.59	92.20	*91.83*	*83.13*	89.83	*82.02*	*82.97*	80.32	88.58	87.02
Int.NP.Sim. (FBX)	*93.23*	*90.68*	*94.32*	*92.13*	85.70	*83.95*	80.78	92.47	*93.49*	*83.57*	*94.82*	*81.98*	*82.90*	83.38	88.96	88.16

(a) Mean accuracy ($\mu Accuracy$) score.

$H(\mu Amb.Red., \mu Rec.)$	EPI	ID	GE	NLPBA	SSC	SGREC	MLEE	GREG	GETM	BI	BT	CES	CNL	I2B2	OSIRIS	μ
Int. [3]	91.03	81.04	88.60	80.81	13.66	60.27	61.66	67.63	0.00	59.09	13.08	37.38	30.50	3.15	0.00	45.86
Int.Gaz. [3]	94.16	86.55	91.09	83.73	56.43	67.10	71.22	77.92	10.43	69.93	13.08	39.54	32.39	3.54	7.69	53.65
Int.Sim. [3]	*96.78*	*92.85*	*93.65*	89.27	78.94	82.80	*88.69*	89.99	53.48	83.68	58.11	49.83	43.99	33.60	37.40	71.54
Int.NP.	90.91	66.23	87.86	82.88	14.81	42.26	48.87	47.71	0.00	51.85	13.08	35.60	12.50	3.15	0.00	39.85
Int.Gaz. (FB)	91.33	80.85	88.79	80.81	20.36	59.48	62.81	68.88	0.00	59.06	13.08	47.26	44.90	3.15	0.00	48.05
Int.Gaz. (FBX)	91.15	80.95	88.79	80.98	15.95	58.70	62.15	68.59	0.00	58.99	13.08	47.26	44.50	3.15	0.00	47.62
Int.NP.Gaz.	94.96	88.95	91.20	86.00	67.44	73.36	78.40	82.82	23.79	77.51	33.33	40.81	33.32	7.32	21.43	60.04
Int.NP.Gaz. (FB)	91.55	81.58	88.84	81.84	21.43	60.46	62.82	69.35	0.00	60.01	13.08	49.16	47.24	3.15	0.00	48.70
Int.NP.Gaz. (FBX)	91.44	81.48	88.84	82.01	21.43	61.61	62.71	68.39	0.00	59.92	13.08	49.15	47.24	3.15	0.00	48.70
Int.Sim. (FB)	94.78	85.94	92.10	83.82	51.04	67.77	69.34	82.89	26.09	76.11	26.09	*62.38*	64.52	15.50	7.69	60.40
Int.Sim. (FBX)	95.20	86.74	92.58	84.48	62.59	69.64	75.31	84.53	30.51	78.66	58.14	*62.98*	65.66	17.18	7.69	64.79
Int.NP.Sim.	96.95	92.98	93.71	90.61	*78.47*	85.10	89.40	91.95	71.79	87.20	*61.08*	51.99	44.74	51.80	52.94	76.05
Int.NP.Sim. (FB)	93.63	86.64	92.03	85.77	59.32	73.83	80.56	82.36	45.56	78.05	27.58	61.44	*66.52*	31.88	13.08	65.22
Int.NP.Sim. (FBX)	94.36	87.28	92.30	87.18	71.00	76.59	82.78	84.47	49.06	80.23	64.84	63.27	67.89	33.58	13.08	69.86

(b) Harmonic mean (H) of mean ambiguity reduction($\mu Amb.Red.$) and recall ($\mu Rec.$).

$\mu AmbiguityReduction$	EPI	ID	GE	NLPBA	SSC	SGREC	MLEE	GREG	GETM	BI	BT	CES	CNL	I2B2	OSIRIS	μ
Int. [3]	83.88	68.40	79.70	68.00	7.33	43.20	44.67	51.11	0.00	42.21	7.00	23.00	18.00	1.60	0.00	35.87
Int.Gaz. [3]	89.25	76.67	83.80	72.25	39.33	50.60	55.47	63.89	5.50	54.19	7.00	24.67	19.33	1.80	4.00	43.18
Int.Sim. [3]	94.56	87.60	88.50	81.25	65.33	71.40	80.90	82.11	36.50	73.27	41.00	33.33	28.33	20.20	23.00	60.49
Int.NP.	83.69	49.60	78.50	71.00	8.00	26.80	32.37	31.33	0.00	35.17	7.00	21.67	6.67	1.60	0.00	30.23
Int.Gaz. (FB)	84.38	68.13	80.00	68.00	11.33	42.40	45.88	52.56	0.00	42.18	7.00	31.00	29.00	1.60	0.00	37.56
Int.Gaz. (FBX)	84.06	68.27	80.00	68.25	8.67	41.60	45.18	52.22	0.00	42.10	7.00	31.00	28.67	1.60	0.00	37.24
Int.NP.Gaz.	90.75	80.67	84.00	75.75	51.00	58.20	64.78	70.78	13.50	64.01	20.00	25.67	20.00	3.80	12.00	48.99
Int.NP.Gaz. (FB)	84.75	69.20	80.10	69.50	12.00	43.40	45.88	53.11	0.00	43.17	7.00	32.67	31.00	1.60	0.00	38.23
Int.NP.Gaz. (FBX)	84.56	69.07	80.10	69.75	12.00	44.60	45.76	52.00	0.00	43.06	7.00	32.67	31.00	1.60	0.00	38.21
Int.Sim. (FB)	90.56	76.00	85.80	72.50	34.33	51.40	53.29	71.00	15.00	62.33	15.00	45.67	48.00	8.40	4.00	48.89
Int.Sim. (FBX)	91.38	77.27	86.70	73.50	45.67	53.60	60.75	73.44	18.00	65.84	41.00	46.33	49.33	9.40	4.00	53.08
Int.NP.Sim.	94.94	87.93	88.60	83.50	64.67	74.80	82.25	85.44	56.00	79.01	44.00	35.33	29.00	35.00	36.00	65.10
Int.NP.Sim. (FB)	88.44	77.13	85.70	75.50	42.33	58.80	68.18	70.22	29.50	65.01	16.00	44.67	50.33	19.00	7.00	53.19
Int.NP.Sim. (FBX)	89.81	78.20	86.20	77.75	55.33	62.40	71.53	73.33	32.50	68.12	48.00	46.67	52.00	20.20	7.00	57.94

(c) Mean ambiguity reduction ($\mu AmbiguityReduction$).

$\mu Recall$	EPI	ID	GE	NLPBA	SSC	SGREC	MLEE	GREG	GETM	BI	BT	CES	CNL	I2B2	OSIRIS	μ
Int. [3]	*99.52*	*99.42*	*99.74*	99.56	99.99	*99.63*	*99.53*	*99.92*	100.00	*98.47*	100.00	*99.68*	99.90	99.76	100.00	*99.67*
Int.Gaz. [3]	99.65	*99.37*	99.77	*99.55*	*99.79*	*99.55*	*99.44*	*99.86*	100.00	*98.56*	100.00	*99.60*	99.83	99.76	100.00	99.65
Int.Sim. [3]	99.11	98.78	99.44	99.05	99.71	98.52	98.14	99.53	100.00	97.55	*99.71*	98.65	98.29	*99.75*	100.00	99.08
Int.NP.	*99.50*	99.64	*99.76*	*99.53*	*99.98*	99.89	99.62	99.98	100.00	98.64	100.00	99.72	100.00	99.76	100.00	99.73
Int.Gaz. (FB)	*99.54*	*99.41*	*99.74*	99.56	*99.95*	*99.63*	*99.55*	*99.90*	100.00	*98.49*	100.00	99.41	99.36	99.76	100.00	99.62
Int.Gaz. (FBX)	*99.54*	*99.41*	*99.74*	*99.55*	*99.98*	*99.65*	*99.56*	*99.90*	100.00	*98.49*	100.00	99.40	99.38	99.76	100.00	99.62
Int.NP.Gaz.	*99.58*	*99.14*	*99.75*	*99.46*	99.53	99.20	*99.27*	*99.79*	100.00	*98.22*	*99.91*	99.53	99.74	99.76	100.00	99.53
Int.NP.Gaz. (FB)	*99.54*	*99.34*	*99.73*	*99.51*	*99.91*	*99.60*	*99.57*	*99.88*	100.00	*98.43*	100.00	99.26	99.22	99.76	100.00	99.58
Int.NP.Gaz. (FBX)	*99.54*	*99.33*	*99.73*	*99.51*	*99.92*	*99.58*	*99.57*	*99.88*	100.00	*98.44*	100.00	99.24	99.21	99.76	100.00	99.58
Int.Sim. (FB)	*99.42*	98.88	99.39	99.32	99.45	99.45	*99.22*	99.56	100.00	97.72	*99.97*	98.39	98.38	*99.74*	100.00	99.26
Int.Sim. (FBX)	*99.36*	98.86	99.31	99.33	99.42	99.36	*99.06*	99.57	100.00	97.68	*99.91*	98.28	98.12	*99.75*	100.00	99.20
Int.NP.Sim.	99.04	98.65	99.45	99.05	*99.75*	98.69	97.91	99.52	100.00	97.28	*99.85*	98.36	97.86	*99.64*	100.00	99.00
Int.NP.Sim. (FB)	*99.46*	98.81	99.37	99.27	99.06	99.20	98.44	99.57	*99.99*	97.63	*99.93*	98.38	98.06	*98.88*	*99.99*	99.07
Int.NP.Sim. (FBX)	*99.40*	98.74	99.33	99.22	99.05	99.15	98.22	99.59	100.00	97.58	*99.88*	98.19	97.76	*99.44*	100.00	99.04

(d) Mean recall ($\mu Recall$).
